# Emodin and physcion alleviate cholestatic liver injury by targeting FXR: hepatoprotective components identified in processed *Polygonum multiflorum* Thunb. using a comprehensive two-dimensional biochromatography system

**DOI:** 10.3389/fphar.2025.1706401

**Published:** 2025-12-19

**Authors:** Zhihui Li, Yanqiu Gu, Jianbo Yang, Shaozhan Wang, Shengnan Li, Panpan Chen, Ru Yao, Fangbin Liu, Ying Wang, Rong Wang, Yongfang Yuan

**Affiliations:** 1 Department of Pharmacy, Shanghai Ninth People’s Hospital, Shanghai Jiao Tong University School of Medicine, Shanghai, China; 2 Institute for Control of Chinese Traditional Medicine and Ethnic Medicine, National Institutes for Food and Drug Control, Beijing, China

**Keywords:** liver injury, traditional Chinese medicine, *Polygonum multiflorum* Thunb., FXR, biochromatography

## Abstract

**Introduction:**

*Polygonum multiflorum* Thunb. (PM) is a representative traditional Chinese medicine (TCM) that exerts different effects in raw and processed forms. The hepatotoxicity of PM is markedly reduced after processing, whereas its hepatoprotective effects are enhanced.

**Purpose:**

This study aimed to establish a novel comprehensive two-dimensional (2D) biochromatography system based on farnesoid X receptor (FXR), which is an important target in cholestatic liver injury (CLI), to investigate the material basis and mechanisms underlying the enhanced hepatoprotection and reduced hepatotoxicity of processed PM (P-PM).

**Methods:**

A comprehensive 2D FXR biochromatography system was established by immobilizing FXR on 3-mercaptopropyltrimethoxysilane (MPTS)-modified silica gel. This system was used to identify the FXR-binding components in raw PM (R-PM) and P-PM. Molecular docking, surface plasmon resonance, and frontal affinity chromatography were used to validate the interactions. The hepatoprotective effects of emodin and physcion were assessed in α-naphthylisothiocyanate (ANIT)-induced CLI mouse models, and an FXR antagonist (Z-guggulsterone) rescue experiment was performed. The expression of FXR signaling-related proteins, including FXR, small heterodimer partner (SHP), bile salt export pump (BSEP), Na+-taurocholate cotransporting polypeptide (NTCP), and inflammatory cytokines, was assessed by Western blotting, real-time quantitative reverse transcription PCR, and immunofluorescence.

**Results:**

The comprehensive 2D FXR biochromatography system successfully identified emodin and physcion as key FXR-binding components, with significantly increased content in P-PM. These components may contribute to the enhanced hepatoprotection and reduced hepatotoxicity of P-PM. *In vivo*, emodin and physcion alleviated ANIT-induced CLI, as evidenced by improved histopathological features and decreased serum levels of liver function markers. Mechanistically, both components upregulated the expression of FXR, BSEP, SHP, and NTCP while suppressing inflammatory cytokine expression. Their hepatoprotective effects and FXR-related upregulation could be disrupted by FXR antagonist Z-guggulsterone. These results suggest that emodin and physcion are key components contributing to the hepatoprotective effects of P-PM, likely through activation of the FXR signaling pathway and suppression of inflammation.

**Conclusion:**

This study established a novel, efficient, rapid, and accurate comprehensive 2D FXR biochromatography system, which is suitable for screening targeted components in TCM, and can be extended to research on other TCMs.

## Graphical Abstract

**Figure:**
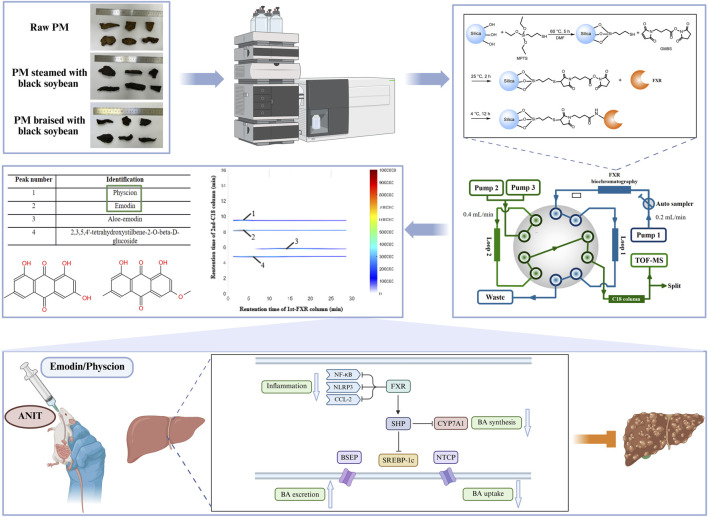

## 1 Introduction

In recent years, liver injury induced by traditional Chinese medicine (TCM) and its preparations has become a major cause of drug induced liver injury (DILI) in clinical practice (Li et al., 2022; Shen et al., 2019; Wang et al., 2022). This poses a substantial obstacle to the clinical application of TCM and the development of new drugs (Zhou et al., 2021). The inherent complexity of TCM, including its diverse chemical constituents, broad targets, and intricate pharmacological mechanisms, makes the investigation of TCM-induced liver injury particularly difficult (Pan et al., 2020). Currently, research methods for TCM-induced liver injury are continuously developing, including *in vitro* cell assays, animal experiments, computer-aided simulations, zebrafish models, and metabolomics approaches (Calitz et al., 2019; He et al., 2019; Jia et al., 2022; Li et al., 2020; Zhang et al., 2022). Calitz et al. (2019) established a 3D co-culture model of HepG2/C3A cells to investigate the potential hepatotoxicity of *Xysmalobium undulatum* aqueous extracts. He et al. (2019) employed computational toxicology to construct an integrated model, screening 98 components of *Polygonum multiflorum* Thunb. (PM), and ultimately identifying 25 hepatotoxic compounds. Li et al. (2020) established a zebrafish hepatotoxicity model to evaluate the hepatotoxicity of 19 compounds of PM, aiming to investigate the material basis of liver injury induced by PM. However, current research methods for DILI are limited by their high cost and long duration, and are not suitable for TCM (Wu et al., 2019). Therefore, developing a novel, efficient, rapid, and accurate technology to identify the material basis of “TCM-liver injury” is crucial for improving the safety assessment of TCM and guiding their clinical application.

Biochromatography is a primary method for screening components that have binding affinity for receptors and investigating drug–receptor interactions (Chen et al., 2013; Hou et al., 2014). This method immobilizes biological materials onto the stationary phase and couples them with highly sensitive detectors such as high-resolution mass spectrometry. It simultaneously enables affinity recognition and compound separation (Moaddel and Wainer, 2009). It has widespread applications in the rapid screening of active components in TCM and the analysis of receptor-ligand interactions (Cao et al., 2018; Wang et al., 2018). In recent years, our team has been continuously optimizing and innovating biochromatographic technologies to better screen active components in TCM (Gu et al., 2019; Gu et al., 2020; Gu D. et al., 2022). Currently, there are no reports of biochromatographic technology being applied in the field of “TCM-liver injury”. Consequently, this study aims to establish a single-target-based comprehensive two-dimensional (2D) biochromatography system to investigate the material basis of “TCM-liver injury”.

PM, a member of the Polygonaceae family, has been used for centuries as a tonic and anti-aging herbal medicine (Lin et al., 2015; Qian et al., 2024). It has been widely used in clinical practice to enhance vitality, strengthen bones, manage alopecia, reduce serum lipid levels, and support liver and kidney function (He et al., 2024; Rao et al., 2021). In recent years, adverse reactions to PM and its preparations, especially reports of liver injury, have drawn significant attention (Teka et al., 2021). Safety issues have now greatly restricted their clinical use (Rao et al., 2021). However, it exerts different effects in both raw and processed forms (Liu et al., 2018; Liu et al., 2019). Raw PM (R-PM) is traditionally used to detoxify and treat malaria, resolve abscesses, and promote bowel movement (Liu et al., 2019). Processed PM (P-PM) is used to tonify the liver and kidneys, strengthen muscles and bones, treat hair loss, and manage premature hair graying (Liu et al., 2018). Numerous studies have demonstrated that the hepatotoxicity of PM is markedly reduced after processing, whereas its hepatoprotective effects are enhanced (Gu D. et al., 2022; Huang et al., 2024; Li et al., 2017; Liu et al., 2019; Xue et al., 2020). Currently, P-PM is included as a constituent in various Chinese patent formulations for treating liver-related disorders, including Yiganning granules (Xue et al., 2020). Several reports have shown that P-PM exhibits hepatoprotective effects, including the prevention of liver injury, and treatment of non-alcoholic fatty liver disease (NAFLD), hepatic fibrosis, and hepatocellular carcinoma (Li et al., 2017; Lin et al., 2020; Xue et al., 2020; Yu et al., 2023). Lin et al. (Lin et al., 2020) reported that P-PM exerted greater efficacy than R-PM in ameliorating NAFLD, likely by enhancing fatty acid β-oxidation. In recent years, increasing attention has been given to elucidating the material basis underlying the hepatoprotective effect of P-PM. The major components contributing to the effect may include anthraquinones and polysaccharides (Wang et al., 2023; Xue et al., 2020). For instance, Wang et al. (2023) reported a significant increase in polysaccharide content following PM processing, and further demonstrated that these polysaccharides exert hepatoprotective effects via antioxidant and anti-inflammatory pathways, providing an explanation for the enhanced hepatoprotection of PM processing. Li J. et al. (2025), Li W. F. et al. (2025) found that the content of dianthrones decreased after PM processing and confirmed that trans-emodin dianthrones can induce significant hepatotoxicity at the cellular level. However, the material basis underlying enhanced efficacy and reduced toxicity following PM processing remains unclear due to its complexity and requires further in-depth investigation.

The clinical characteristics of PM-induced liver injury are predominantly cholestatic and hepatocellular types (Dong et al., 2014; Wang et al., 2019). Farnesoid X receptor (FXR) is one of the most important targets in cholestatic liver injury (CLI) (Petrescu and DeMorrow, 2021; Trauner and Fuchs, 2022). FXR, a nuclear receptor highly expressed in the liver and intestine, is activated by bile acids (BAs) (Ding et al., 2015). It serves as a key regulator of BA biosynthesis and enterohepatic circulation, and plays a vital role in maintaining BA homeostasis (Stofan and Guo, 2020). It modulates the expression of genes and proteins involved in BA synthesis and transport (Adorini and Trauner, 2023; Perino et al., 2021). FXR inhibition induces the dysregulation of these proteins, thereby impeding bile flow and triggering intrahepatic cholestasis (Guo et al., 2016). Conversely, activation of FXR can ameliorate CLI (Zeng et al., 2023). In hepatocytes, FXR activation induces the expression of the small heterodimer partner (SHP), which subsequently suppresses cytochrome P450 7A1 (CYP7A1) expression, thereby reducing BA synthesis (Yan et al., 2021; Zeng et al., 2023). Furthermore, FXR regulates the expression of BA transporters such as bile salt export pump (BSEP), multidrug resistance-associated protein 2 (MRP2), and Na^+^-taurocholate cotransporting polypeptide (NTCP) (Panzitt and Wagner, 2021). These changes inhibit BA uptake and enhance its excretion, ultimately relieving intrahepatic cholestasis (Adorini and Trauner, 2023; Panzitt and Wagner, 2021). In conclusion, FXR is a critical target for CLI.

Therefore, this study established a comprehensive 2D FXR biochromatography system using PM as a model, and FXR as the target. The FXR protein was covalently immobilized onto 3-mercaptopropyltrimethoxysilane (MPTS)-modified silica gel surfaces through aldehyde groups, producing an FXR biochromatographic stationary phase with defined content and stable conformation. In this study, we established a novel MPTS-modified comprehensive 2D FXR biochromatography system. Following systematic methodological validation, the technology was applied to identify components in both R-PM and P-PM that have binding affinity for FXR. Subsequently, the binding interactions between these components and FXR were verified using techniques such as surface plasmon resonance (SPR). Finally, *in vivo* studies evaluated the roles of key compounds in the hepatoprotective effect of P-PM and elucidated the mechanisms underlying its enhanced hepatoprotection and reduced hepatotoxicity following processing. In conclusion, a comprehensive 2D FXR biochromatography system was established to identify the FXR-affinity compounds of R-PM and P-PM. This technology helps clarify the material basis and mechanisms through which P-PM targets FXR to alleviate CLI.

## 2 Methods

### 2.1 Reagents

R-PM, PM steamed with black soybean juice (S-BSJ-PM), and PM stewed with black soybean juice (T-BSJ-PM) were provided by the National Institutes for Food and Drug Control (Beijing, China). Emodin (purity ≥98%; Cat: 12,000–2,401), physcion (purity ≥98%; Cat: 040021-202403) and Z-guggulsterone (Z-GS, purity ≥98%; Cat: 12956-2501) were obtained from Shanghai Chishao Biotechnology Co., Ltd. (Shanghai, China). 2, 3, 5, 4′-tetrahydroxystilbene-2-O-β-D-glucoside (TSG, purity ≥98%; Cat: B21757) and aloe-emodin (purity ≥98%; Cat: B20772) were obtained from Yichu Biotechnology Co., Ltd. (Shanghai, China). GW4064 (purity >99.5%; Cat: HY-50108) was purchased from MedChemExpress (Monmouth Junction, NJ, United States). α-Naphthylisothiocyanate (ANIT, purity ≥98%; Cat: N106389), ursodeoxycholic acid (UDCA, purity ≥99%; Cat: U110695), and obeticholic acid (purity ≥98%; Cat: I193491) were purchased from Aladdin (Shanghai, China). The human FXR protein (His Tag; Cat: 17624-H07E) was purchased from Sino Biological (Beijing, China). Primary antibody against FXR (Cat: 680469) was purchased from Chengdu Zhengneng Biotechnology Co., Ltd. (Chengdu, China). Anti-SHP antibody (Cat: bs-4311R) was obtained from Beijing Bioss Biotechnology Co., Ltd. (Beijing, China). Anti-NTCP (Cat: A06872-1) and anti-BSEP (Cat: PB9414) antibodies were obtained from Boster Biological Technology Co., Ltd. (Wuhan, China). The β-actin antibody (Cat: 4967S) and HRP-conjugated secondary antibody against rabbit IgG (Cat: 7074P2) were obtained from Cell Signaling Technology (Danvers, MA, United States).

### 2.2 Preparation of PM extracts

R-PM, S-BSJ-PM and T-BSJ-PM were pulverized to powder samples. Samples (5 g) were soaked in 50 mL of 70% ethanol (v/v) overnight and followed by ultrasonic-assisted reflux extraction (2 h). After centrifugation at 3,000 g for 20 min, supernatants were collected. The remaining residue underwent a second extraction using identical methods. The two extracts were then combined. The combined extracts were concentrated to dryness by rotary evaporation, and the residue was accurately weighed. The dried extract was then dissolved in solvent to obtain a final concentration of 1 g/mL. The resulting solution was stored at 4 °C until future application.

### 2.3 Preparation of FXR biochromatographic stationary phase

The preparation of the biochromatographic stationary phase was based on our previous research (Gu et al., 2020). Silica gel (1 g, 5 μm, 200 Å) was added to MPTS (1 mL) and stirred for 5 h in DMF (100 mL) at 60 °C under nitrogen atmosphere. The mixture was centrifuged at 5,000 g and washed three times with DMF. The precipitate was reacted with 5% N-(4-maleimide butyryl oxide) succinimide (GMBS) in 500 mL DMSO solution and stirred for 2 h. After washing with DMSO, the modified silica gel was dried for 48 h. 40 mg modified silica gel was activated at 120 °C for 1 h. Then, in a fritted tube, 20 μg of the FXR protein solution was reacted with the silica gel for 5 min at 4 °C under vacuum and vortex conditions. The mixture was magnetically stirred for 45 min at 4 °C and then incubated overnight. After washing and resuspending in PBS, the FXR stationary phase was packed into a chromatography column using wet-packing with PBS buffer on a Waters 996–515 pump. The flow rate was increased from 0.2 mL/min to 1.0 mL/min over 5 min, followed by equilibration at 0.2 mL/min for 30 min until a stable pressure was achieved.

### 2.4 Construction of comprehensive 2D FXR biochromatography and component screening

An Agilent 1290 HPLC system coupled with a 6220 TOF mass spectrometry was used to establish a comprehensive 2D FXR biochromatography system. An FXR column (10 × 2 mm i.d., 5 μm) served as the first dimension (1st). An Agilent Poroshell 120 EC-C18 column (150 × 3.0 mm i.d., 2.7 μm) was used as the second dimension (2nd). The sample injection volume was 5 μL. The flow rates were set to 0.2 mL/min for the 1st and 0.4 mL/min for the 2nd. The 1st mobile phase was 10 mM ammonium acetate; the 2nd mobile phase consisted of 0.1% formic acid-water and acetonitrile. The mobile phase consisted of solvent A (0.1% formic acid in water) and solvent B (0.1% formic acid in acetonitrile). A gradient elution was applied as follows: 0–2 min, 95% A; 2–13 min, 95%–5% A; 13–15 min, 5% A. Correspondingly, solvent B changed from 5% at 0–2 min, 5%–95% at 2–13 min, and 95% at 13–15 min. The prepared FXR column was integrated into the system. The extracts of R-PM, S-BSJ-PM, and T-BSJ-PM were injected and subjected to automated analysis. In 1st, samples were passed through the FXR column to screen for potential compounds. A 10-port two-position valve (Valve 2) was switched every 2.5 min. Eluents from the 1st column were sequentially enter into the 2nd column for analysis in sequence according to different elution times. Mass Hunter software was used to collect retention behavior and molecular structure information, enabling the identification of the specific constituents. The data were imported into MATLAB 7.10.0 for generation of 2D retention contour maps.

### 2.5 SPR analysis

Emodin, physcion, and GW4064 were dissolved in DMSO to prepare 10 mM stock solutions. SPR experiments were conducted on a Biacore T200 system using a GM5 sensor chip. The chip surface was first activated for 420 s, followed by the injection of the FXR protein solution for covalent immobilization over 600 s. After coupling, excess active sites were blocked by injecting ethanolamine for 420 s. The coupling buffer was 1 × PBS and the flow rate during the coupling process was maintained at 10 μL/min. Stock solutions of emodin, physcion, and GW4064 were diluted with a running buffer containing 5% DMSO to obtain a series of concentration gradients. Each sample was injected at a flow rate of 30 μL/min for 60 s, followed by a dissociation phase of 180 s. The sensorgrams were analyzed using the Biacore T200 Evaluation Software.

### 2.6 Frontal affinity chromatography (FAC)

Emodin and physcion solutions of different concentrations were prepared using PBS as the mobile phase for later use. The FXR column was prepared as described previously and installed on the screening system. The FXR column was equilibrated with PBS for 20 min at a flow rate of 1 μL/min. Subsequently, the mobile phase was replaced with PBS containing 1 μM of emodin and physcion. The baseline was monitored until breakthrough curves appeared, and the breakthrough time was recorded. After the baseline returned to stability, the mobile phase was switched back to PBS to re-equilibrate the column. Thereafter, the mobile phase was replaced with PBS containing 5, 10, 25, 50, and 100 μM of emodin and physcion to obtain the breakthrough times for six concentrations. The dissociation constants (K_D_) of emodin and physcion were calculated.

### 2.7 Molecular docking

Molecular docking was conducted using AutoDock-GPU1 to predict the potential conformations and orientations of small molecules at binding sites. The crystal structure of FXR was downloaded from RCSB Protein Data Bank under PDB code 6HL1 and processed by adding hydrogen atoms and eliminating water molecules to prepare it as the receptor. The three-dimensional (3D) molecular structures of emodin, physcion, and GW4064 were retrieved from PubChem and used as docking ligands. The Lamarckian Genetic Algorithm and empirical free energy scoring function were used for docking simulation. The binding pocket of FXR was created based on the co-crystal ligand in the crystal structure. The grid center (x = 10.847, y = −15.079, z = 12.613 in Å) and dimensions 30 × 30 × 30 Å were used as the grid parameters for docking simulation. Each ligand underwent 100 genetic algorithms (GA) in each docking simulation. The docking pose with the lowest energy score was chosen as the most likely binding complex of small molecules and FXR. The interactions between small molecules and FXR were visualized using Discovery Studio Visualizer, while the 3D binding models of small molecules and FXR were mapped using PyMOL software.

### 2.8 Animal experiments

Emodin/Physcion: Male C57BL/6 mice (7–9 weeks old) were randomly divided into 6 groups (N = 8): (1) control, (2) model, (3) positive control (UDCA 60 mg/kg), (4) low-dose emodin/physcion (20 mg/kg), (5) high-dose emodin/physcion (40 mg/kg), and (6) emodin/physcion-alone (40 mg/kg). The administration volume of all groups was 0.1 mL/10 g. Emodin, physcion, and UDCA were formulated as suspensions in 0.5% sodium carboxymethyl cellulose (CMC-Na). ANIT was dissolved in the peanut oil. Mice in the control and model groups received 0.5% CMC-Na orally once daily for 7 days. On day 5, the control group was given peanut oil, whereas the model group was orally administered 60 mg/kg ANIT to induce CLI. For seven consecutive days, mice in the low- and high-dose emodin/physcion groups were administered emodin/physcion orally once daily at dosages of 20 and 40 mg/kg, respectively. Mice in the positive drug group received an oral dose of 60 mg/kg UDCA daily for 7 days. The emodin/physcion-alone group received 40 mg/kg emodin/physcion alone daily for 7 days. All groups, except the control and emodin/physcion-alone groups, were orally administered 60 mg/kg ANIT on day 5. All the mice were euthanized on day 8. Body and liver weights were recorded, and orbital blood was collected. Serum samples and a portion of the liver tissue were frozen at −80 °C, whereas the remaining tissues were fixed in 4% paraformaldehyde for histopathological examination.

### 2.9 Serum biochemical analysis

Orbital blood samples were centrifuged at 4 °C and 3,000 × g for 15 min to obtain serum. Serum biochemical indicators, including direct bilirubin (DBIL), total bile acid (TBA), alanine aminotransferase (ALT), and aspartate aminotransferase (AST) were measured using an automated biochemical analyzer.

### 2.10 Hematoxylin–eosin (H&E) staining

Liver tissues fixed in 4% paraformaldehyde were embedded in paraffin and sectioned. These sections were stained with hematoxylin and eosin to evaluate histopathological changes under a light microscope.

### 2.11 Western blotting (WB)

Protein samples were separated by SDS-PAGE. After separation, the proteins were transferred onto PVDF membranes. Membranes were blocked using a rapid blocking buffer on a shaker for 30 min. Primary antibodies (FXR, SHP, NTCP, and BSEP) were added and incubated overnight at 4 °C, followed by secondary antibodies at 25 °C for 1 h. Protein bands were visualized using an Affinity™ ECL kit (Femtogram), and imaged using a ChemiDoc XRS + imaging system. Quantitative analysis of band intensity was performed using ImageJ software.

### 2.12 Quantitative real-time polymerase chain reaction (qRT-PCR)

Total RNA was extracted from liver tissues using TRIzol™ reagent. Then RNA concentration was measured using a NanoDrop spectrophotometer and diluted to an appropriate concentration. Primer sequences are provided in Supplementary Table S7. The primers were diluted to a comcentration of 10 μM. Reverse transcription and amplification were performed using the one-step RT-PCR kit. Amplification and melting curves were analyzed, and Ct values were obtained for relative quantification.

### 2.13 Immunofluorescence staining

Liver tissues were embedded in paraffin and sectioned. After dewaxing and rehydration, antigen retrieval was performed on these sections. These sections were blocked with 3% BSA for 30 min, followed by incubation with primary antibodies overnight at 4 °C. Then sections were incubated with secondary antibodies for 1 h in the dark after washing. Nuclei were stained with DAPI, and the sections were mounted. Fluorescence images were captured using a fluorescence microscope.

### 2.14 Statistical analysis

GraphPad Prism 9 was used for statistical analysis. For datasets confirming to normal distribution and homogeneity of variance, one-way ANOVA was performed to analyze differences among multiple groups, while Student’s t-test was applied for comparisons between two groups. Results were reported as mean ± standard deviation (SD), and statistical significance was defined as *P* < 0.05. The graphical abstract was created using BioRender.

## 3 Results

### 3.1 Construction of comprehensive 2D FXR biochromatography system and component screening

**FIGURE 1:**
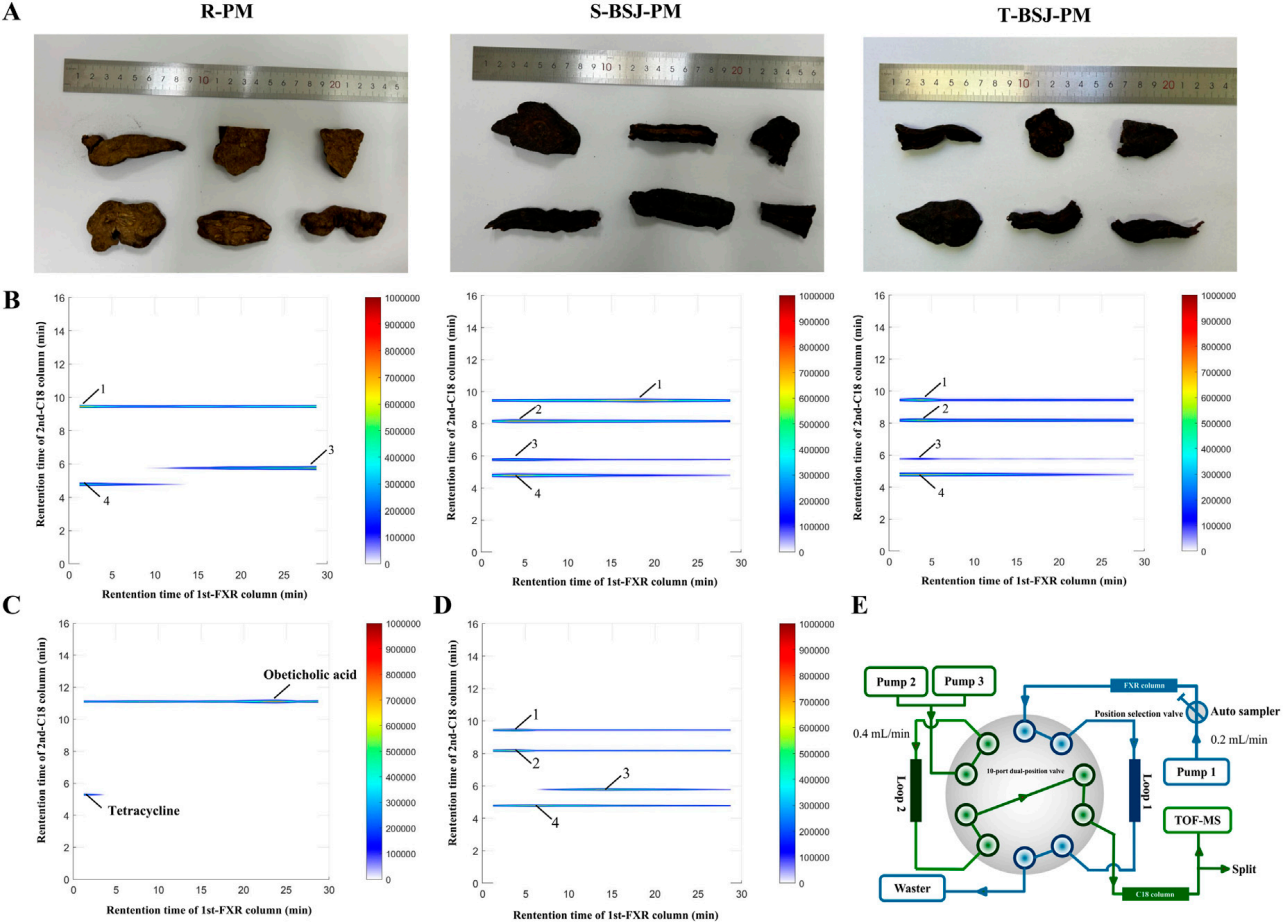
Construction of the MPTS-modified comprehensive 2D FXR biochromatography system and component screening. **(A)** Images of the R-PM, S-BSJ-PM, and T-BSJ-PM samples. **(B)** Typical 2D plots of R-PM, S-BSJ-PM, and T-BSJ-PM extracts obtained using the comprehensive 2D FXR biochromatography system. **(C)** 2D plots of obeticholic acid and tetracycline obtained using a comprehensive 2D FXR biochromatography system. **(D)** 2D plots of emodin, physcion, aloe-emodin, and TSG standards obtained using a comprehensive 2D FXR biochromatography system. **(E)** Diagram of the comprehensive 2D FXR biochromatography system.

To investigate the material basis of enhanced hepatoprotection and reduced hepatotoxicity after PM processing, three representative samples were selected: R-PM, S-BSJ-PM, and T-BSJ-PM (Figure 1A). This study established an MPTS-modified comprehensive 2D FXR biochromatography system (Figure 1E). Obeticholic acid (a positive control drug) and tetracycline (a negative control drug) were used to evaluate the effectiveness and specificity of the system. As shown in Figure 1C, obeticholic acid exhibited strong retention on the FXR column, with a peak time of approximately 22.5 min, indicating its binding to FXR. In contrast, tetracycline eluted near the dead time without retention, suggesting no observable binding to FXR. These results confirmed that the comprehensive 2D FXR biochromatography system possessed good effectiveness and selectivity, making it suitable for subsequent component screening. After validating its performance, the technology was applied to screen potential components that bind to FXR from PM extracts. As shown in Figure 1B and Table 1, emodin, physcion, aloe-emodin, and TSG exhibited significant retention on the FXR column, indicating their potential binding to FXR. To further validate these interactions, the retention behavior of standards was examined using this system. As shown in Figure 1D, emodin, physcion, aloe-emodin, and TSG exhibited distinct retentions, thereby confirming their strong binding affinities for FXR.

**TABLE 1:** Potential components in R-PM, S-BSJ-PM, and T-BSJ-PM characterized by comprehensive 2D FXR biochromatography system.

Sample	Number	Identification	tR (C18,min)	tR (FXR,min)	Quasi-molecular ion	m/z	Error (ppm)	Abund match (%)	Fomula
R-PM	1	Physcion	9.45	2.5–30	[M + H]+	285.0762	1.47	98.98	C_16_H_12_O_5_
R-PM	3	Aloe-emodin	5.78	12.5–30	[M + H]+	271.0607	−0.33	84.94	C_15_H_10_O_5_
R-PM	4	TSG	4.79	2.5–12.5	[M + H]+	407.1332	−1.64	97.96	C_20_H_22_O_9_
S-BSJ-PM	1	Physcion	9.45	2.5–30	[M + H]+	285.0764	1.83	98.25	C_16_H_12_O_5_
S-BSJ-PM	2	Emodin	8.18	2.5–30	[M + H]+	271.0616	5.15	92.17	C_15_H_10_O_5_
S-BSJ-PM	3	Aloe-emodin	5.78	2.5–30	[M + H]+	271.0607	0.61	84.99	C_15_H_10_O_5_
S-BSJ-PM	4	TSG	4.79	2.5–27.5	[M + H]+	407.1343	1.09	98.6	C_20_H_22_O_9_
T-BSJ-PM	1	Physcion	9.45	2.5–30	[M + H]+	285.0763	1.44	98.44	C_16_H_12_O_5_
T-BSJ-PM	2	Emodin	8.18	2.5–30	[M + H]+	271.0610	3.06	96.61	C_15_H_10_O_5_
T-BSJ-PM	3	Aloe-emodin	5.78	2.5–30	[M + H]+	271.0604	1.23	85.76	C_15_H_10_O_5_
T-BSJ-PM	4	THSG	4.79	2.5–27.5	[M + H]+	407.1347	2.19	96.9	C_20_H_22_O_9_

### 3.2 Effect of processing on the content of emodin and physcion in PM

**FIGURE 2:**
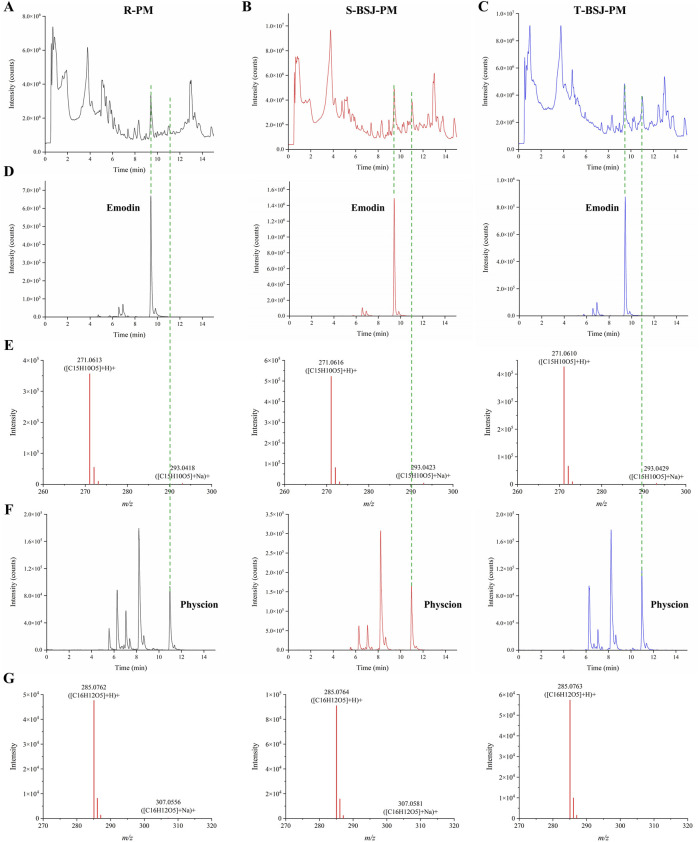
UPLC-QTOF/MS analysis of emodin and physcion in R-PM, S-BSJ-PM, and T-BSJ-PM. **(A–C)** Total ion chromatograms (TICs) of R-PM, S-BSJ-PM, and T-BSJ-PM extracts detected by using UPLC-QTOF/MS. **(D,E)** Extracted ion chromatograms (EICs, D) and MS spectra **(E)** of emodin in different PM extracts. **(F,G)** EICs **(F)** and MS spectra **(G)** of physcion in different PM extracts.

Previous studies have shown that processing reduces the hepatotoxicity of PM while enhancing its hepatoprotective effects, which may be related to the increased content of free anthraquinones and polysaccharides in the P-PM (Li et al., 2017; Xue et al., 2020; Wang et al., 2023). Therefore, Ultra-Performance Liquid Chromatography Quadrupole Time-of-Flight Mass Spectrometry (UPLC-QTOF/MS) was employed to identify the chemical constituents of R-PM, S-BSJ-PM, and T-BSJ-PM (Figures 2A–C). Compared to R-PM, both S-BSJ-PM and T-BSJ-PM showed significantly increased peak areas of emodin and physcion, indicating that the content of these compounds increased markedly after processing (Figures 2D,F). Moreover, the identities of emodin and physcion were confirmed by their primary MS spectra, which were consistent with the calculated exact masses and previous reports (Figures 2E,G). To quantitatively validate these observations, the concentrations of emodin and physcion were further determined using High-Performance Liquid Chromatography (HPLC) (Supplementary Figure S1). Linear regression equations for both compounds showed excellent linearity within the tested ranges (Supplementary Table S1). Quantitative results confirmed that the concentrations of emodin and physcion were significantly higher in the S-BSJ-PM and T-BSJ-PM than in the R-PM (Supplementary Table S2, *P* < 0.05). Given that emodin and physcion have been widely reported to exert hepatoprotective effects against liver injury (Bai et al., 2025; Chen et al., 2023; Xie et al., 2019; Yao et al., 2020), they may be the key components responsible for the hepatoprotective effect of P-PM.

### 3.3 Binding of emodin and physcion to FXR

**FIGURE 3:**
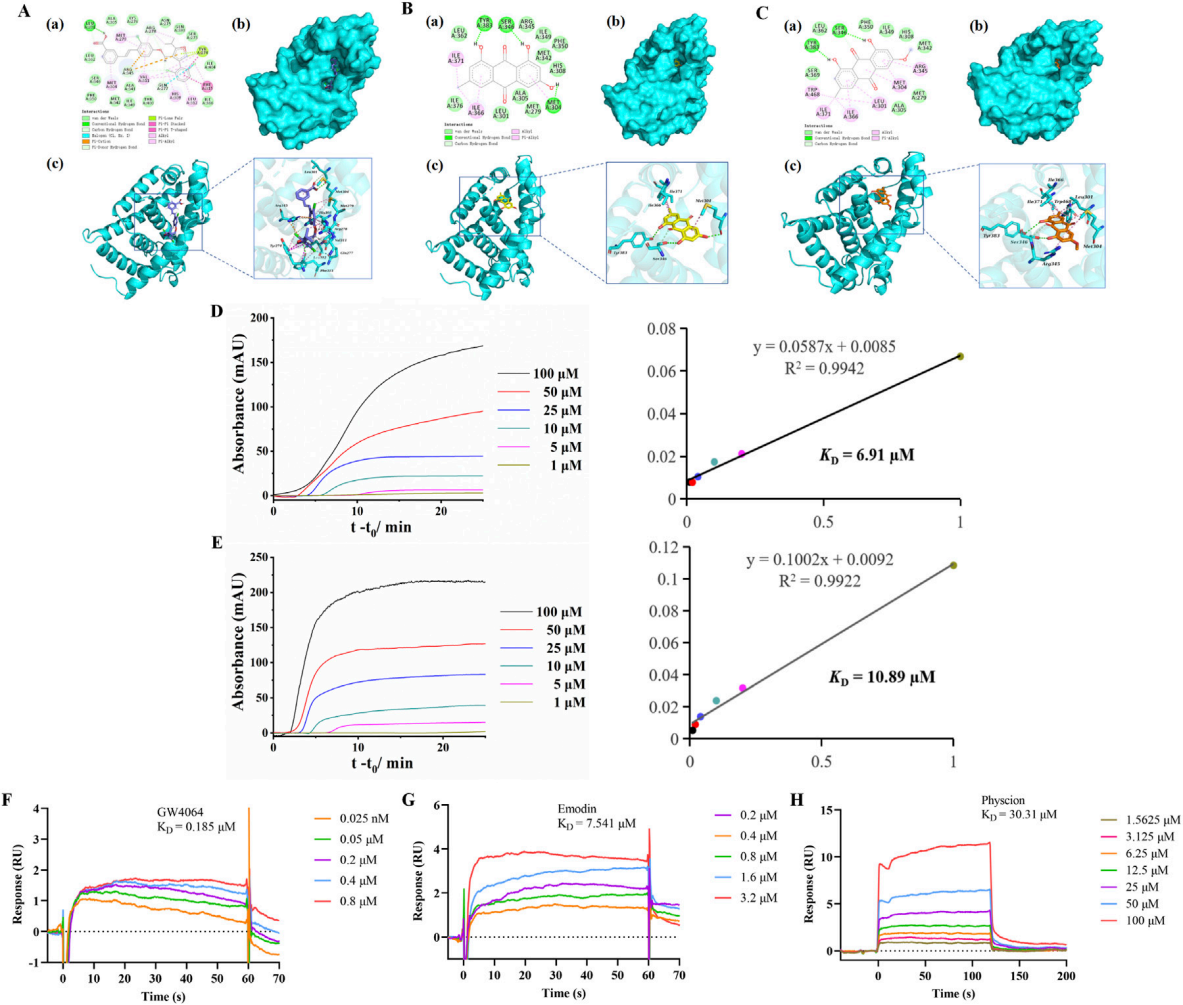
Binding of emodin and physcion to FXR. **(A–C)** Predicted binding modes of GW4064 (A, slate), emodin (B, yellow), and physcion (C, orange) in the FXR (cyan) binding pocket. **(a)** 2D binding mode. **(b,c)** 3D binding mode. **(D,E)** FAC for determining the K_D_ of emodin **(D)** and physcion **(E)** on FXR columns: breakthrough curves and nonlinear fitting curves. **(F–H)** SPR analysis of GW4064 **(F)**, emodin **(G)**, and physcion **(H)**.

To further evaluate the binding affinity of emodin and physcion for FXR, molecular docking was performed using GW4064, a known FXR agonist, as a reference. GW4064 showed a docking score of −16.98 kcal/mol and showed suitable steric complementarity with the binding site of FXR (Figure 3A). Its binding was stabilized by multiple interactions, including hydrogen bonds, halogen interactions electrostatic interactions, pi-lone pair interactions, hydrophobic interactions and Van der Waals (VDW), particularly involving residues Leu301, Arg345, Met304, Arg278, Tyr274, Phe315, and Phe350.

Emodin and physcion showed docking scores of −7.04 kcal/mol and −6.83 kcal/mol, respectively. Both compounds exhibited good steric complementarity with the binding site of FXR (Figures 3B,C). Emodin and physcion formed conventional hydrogen bonds with key residues such as Ser346, and Tyr383, along with hydrophobic and VDW interactions with Leu301, Arg345, Met304, Ile366 and Phe350. These interactions indicate that emodin and physcion share a comparable FXR binding mode with GW4064, supporting their potential as FXR-targeting compounds.

Furthermore, FAC was employed to evaluate the interactions between FXR and two components. As shown in Figures 3D,E, the breakthrough curves of emodin and physcion at various concentrations demonstrated a clear trend: with decreasing concentrations, the breakthrough times increased. The K_D_ values of emodin and physcion were calculated to be 6.91 μM and 10.89 μM, respectively. These results indicate that emodin and physcion have strong affinities for FXR. SPR assays were performed to validate the affinity of the two components for FXR. GW4064 was used to study the validity and selectivity of the SPR system. The K_D_ value of GW4064 was 0.19 μM, confirming its strong binding affinity for FXR and demonstrating the validity and selectivity of the SPR system (Figure 3F). As shown in Figures 3G,H, the K_D_ values of emodin and physcion were 7.54 μM and 30.31 μM, respectively. These results support the conclusion that emodin and physcion exhibit strong binding affinities for FXR.

### 3.4 Effect of emodin on ANIT-induced CLI and safety in mice

**FIGURE 4:**
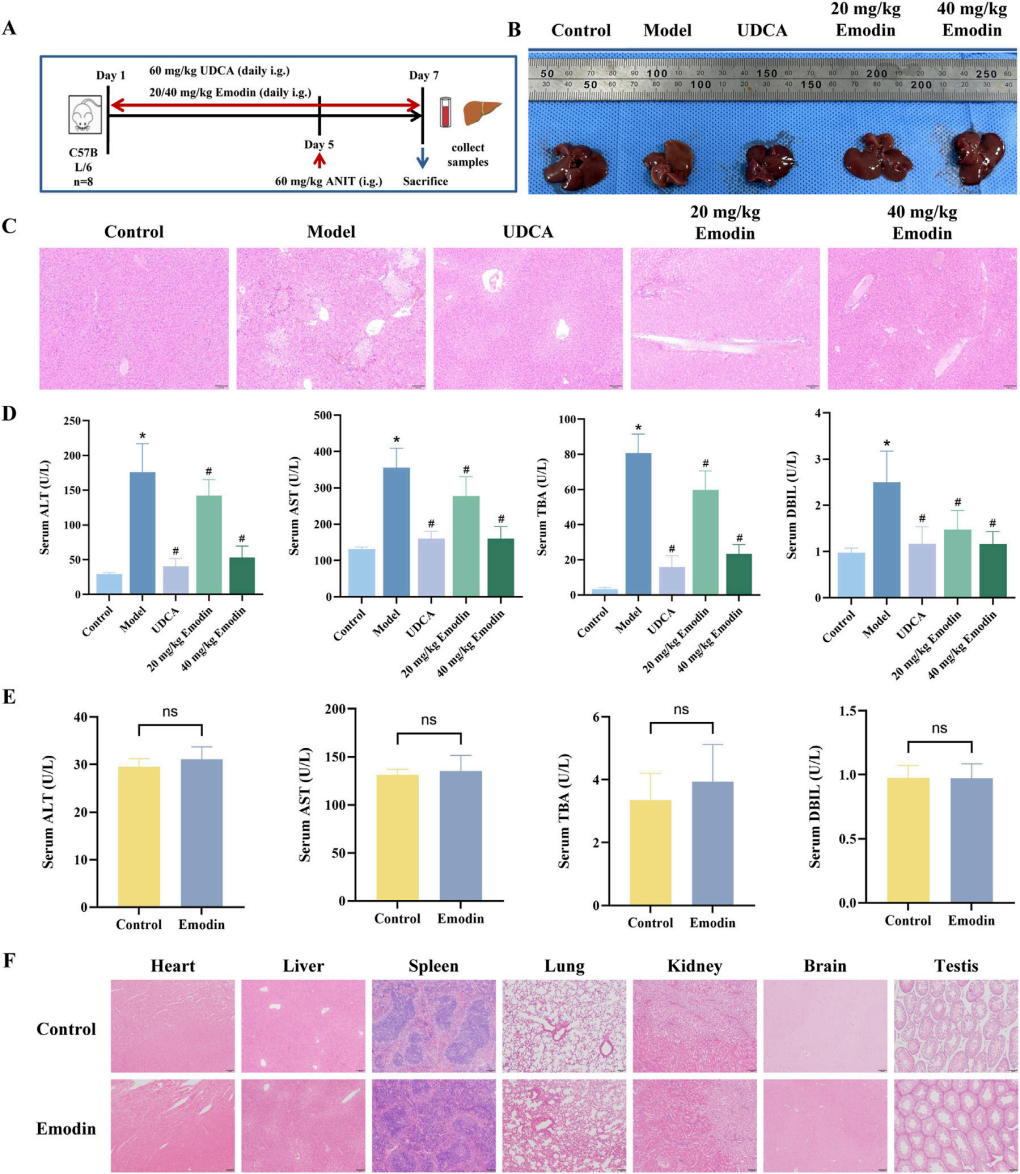
Effect of emodin on ANIT-induced CLI and safety in mice **(A)** Experimental scheme for ANIT-induced CLI and emodin treatment in mice. **(B)** Macroscopic appearance of livers from each group. **(C)** Representative images of HE-stained liver sections from each group of mice. **(D)** Serum levels of ALT, AST, TBA, and DBIL. N = 8. **(E)** Serum levels of ALT, AST, TBA, and DBIL after administration of emodin alone at 40 mg/kg. N = 8. **(F)** Histopathological assessment of major organs after emodin administration alone. All data are presented as mean ± SD. *P < 0.05 vs. Control, #*P* < 0.05 vs. Model, ns *P* > 0.05 vs. Control.

The experimental procedures for establishing the CLI mouse model and emodin administration are summarized in Figure 4A. As shown in Figure 4B, livers from the control group exhibited normal morphology, reddish-brown color, and smooth surfaces, whereas those from the model group appeared yellow, with grayish-white spots on the surface and fragile margins. In contrast, the UDCA and emodin-treated groups showed notable improvements in liver appearance. Histological analysis revealed that the hepatic lobule structure was clear and intact, with normal central veins and portal areas in the control group (Figure 4C). Hepatocytes were arranged radially around the central veins. In contrast, the model group exhibited widespread hepatocyte necrosis, edema accompanied by inflammatory infiltration, and congestion in the portal areas. These pathological changes were significantly alleviated by UDCA and emodin treatment. Serum levels of ALT, AST, TBA, and DBIL were markedly elevated in the model group but significantly reduced in the UDCA and both low- and high-dose emodin groups (Figure 4D, *P* < 0.05), with detailed percentage reductions summarized in Supplementary Table S3. These results indicate that emodin exerts hepatoprotective effects against ANIT-induced CLI.

In addition, the safety of emodin was preliminarily evaluated by administering 40 mg/kg emodin alone to mice. As shown in Figure 4E, there were no significant differences in the serum levels of ALT, AST, TBA, and DBIL between the emodin-alone and control groups (*P* > 0.05). HE staining results revealed no observable pathological abnormalities in major organs, including the heart, liver, spleen, lung, kidney, brain, and testis (Figure 4F).

### 3.5 Regulation of inflammatory factors and the FXR signaling pathway by emodin in CLI mice

**FIGURE 5:**
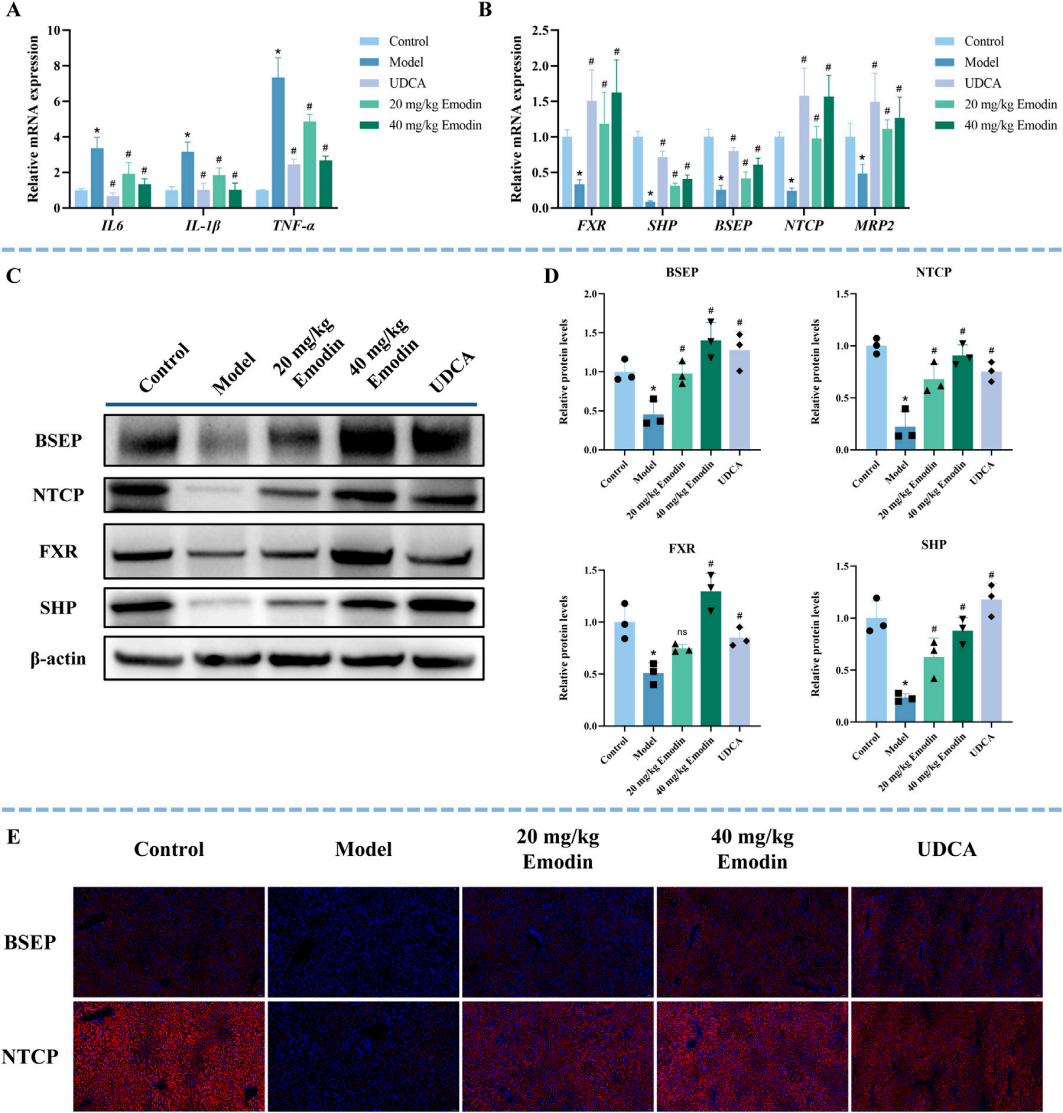
Effect of emodin on inflammatory factors and the FXR signaling pathway in ANIT-induced CLI mice. **(A)** The mRNA expression of inflammatory factors (*IL-6*, *IL-1β*, and *TNFα*) was determined by qRT-PCR. N = 5. **(B)** The mRNA expression of *FXR, SHP*, *NTCP*, *BSEP*, and *MRP2* was determined using qRT-PCR. N = 5. **(C,D)** Representative WB images and quantitative analysis of FXR, SHP, BSEP, and NTCP protein expression in liver tissues. N = 3 independent biological replicates per group. **(E)** Representative immunofluorescence images of BSEP and NTCP (red) in the liver sections. Nuclei are counterstained with DAPI (blue). All data are presented as mean ± SD. *P < 0.05 vs. Control, #*P* < 0.05 vs. Model, ns *P* > 0.05 vs. Model.

CLI involves the release of multiple inflammatory factors, and the sustained stimulation of these mediators aggravates liver injury (Zhuang et al., 2024). As shown in Figure 5A, the mRNA expression levels of interleukin-6 (*IL-6*), interleukin-1 beta (*IL-1β*), and tumor necrosis factor alpha (*TNFα*) were markedly elevated in the model group (*P* < 0.05), while UDCA and emodin treatment significantly reduced their expression in a dose-dependent manner (*P* < 0.05).

The FXR signaling pathway is a key pathway in CLI (Wang et al., 2024). To further explore the effect of emodin on FXR and its downstream targets, this study used qRT-PCR, WB, and immunofluorescence to study the expression of FXR, SHP, BSEP, and NTCP. Compared with the control group, the mRNA expression levels of *FXR*, *SHP*, *BSEP*, *NTCP*, and *MRP2* were significantly decreased in the model group, whereas UDCA and emodin treatment significantly upregulated their expression (Figure 5B, *P* < 0.05). The quantitative fold changes relative to the model group are summarized in Supplementary Table S5. Consistently, WB results showed that the expression of FXR, SHP, BSEP, and NTCP was significantly suppressed in the model group (Figures 5C,D, *P* < 0.05). In contrast, treatment with UDCA and both low- and high-dose emodin markedly restored the expression of SHP, BSEP, and NTCP (*P* < 0.05). FXR expression was also significantly upregulated in the UDCA and high-dose emodin groups (*P* < 0.05), but not in the low-dose emodin group, where only a non-significant increase was observed (*P* > 0.05). Immunofluorescence results further confirmed that the expression of BSEP and NTCP was significantly reduced in the model group and their dose-dependent restoration was observed after UDCA and emodin treatment (Figure 5E). These results suggest that emodin alleviates CLI by suppressing the expression of inflammatory factors and activating the FXR signaling pathway.

### 3.6 Effect of FXR inhibition on the hepatoprotective activity of emodin in ANIT-induced CLI

**FIGURE 6:**
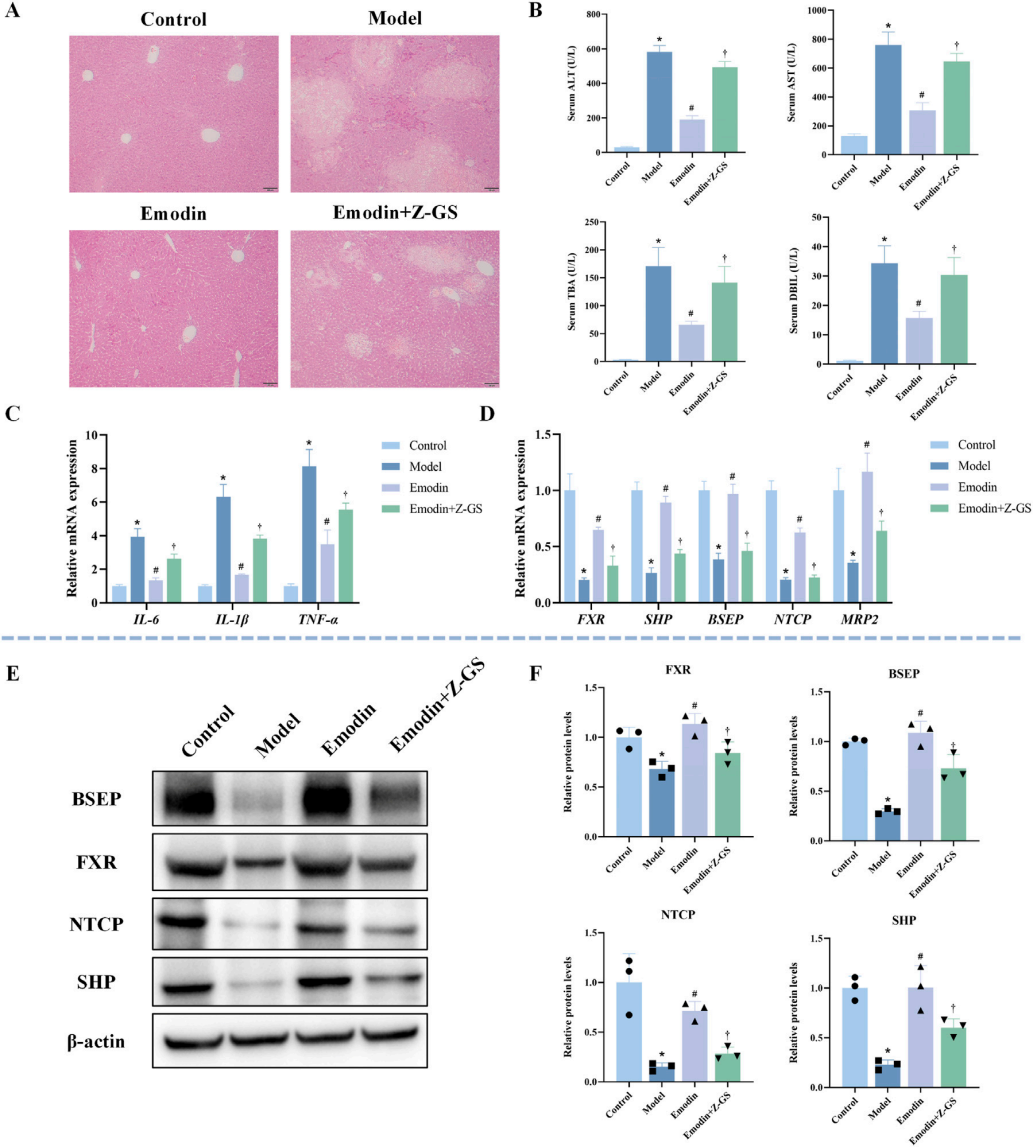
Impact of FXR inhibition on emodin-mediated hepatoprotection and FXR pathway activation in ANIT-induced CLI mice **(A)** Representative images of HE-stained liver sections. **(B)** Serum levels of ALT, AST, TBA, and DBIL. N = 8. **(C)** Hepatic mRNA expression of inflammatory cytokines (*IL-1β*, *IL-6*, and *TNF-α*) was quantified by qRT-PCR. N = 3. **(D)** The mRNA expression of *FXR* and its target genes (*SHP*, *BSEP*, *NTCP*, and *MRP2*) was quantified by qRT-PCR. N = 3. **(E,F)** Representative WB images and quantitative analysis of FXR, SHP, BSEP, and NTCP protein expression in liver tissues. N = 3 independent biological replicates per group. Data are presented as the mean ± SD. *P < 0.05 vs. Control; #*P* < 0.05 vs. Model; †*P* < 0.05 vs. Emodin.

To verify whether the hepatoprotective effect of emodin depends on the activation of FXR signaling, the FXR antagonist Z-GS was co-administered with emodin (40 mg/kg) in CLI mice. As shown in Figure 6A, co-administration of Z-GS markedly attenuated the protective effect of emodin on liver histopathological injury. Consistently, Z-GS significantly diminished the ability of emodin to reduce serum levels of ALT, AST, TBA, and DBIL (Figure 6B). In addition, Z-GS notably reversed the inhibitory effects of emodin on pro-inflammatory cytokines, including *IL-1β*, *IL-6*, and *TNF-α* (Figure 6C). At the molecular level, Z-GS treatment significantly attenuated the emodin-induced upregulation of *FXR* and its downstream targets *SHP*, *BSEP*, *NTCP*, and *MRP2* (Figure 6D). WB analysis further confirmed that the enhanced expression of FXR, SHP, BSEP, and NTCP following emodin treatment was significantly reduced upon Z-GS co-administration (Figures 6E,F). These results indicate that the hepatoprotective effect of emodin against CLI is mediated through FXR activation.

### 3.7 Effect of physcion on ANIT-induced CLI and safety in mice

**FIGURE 7:**
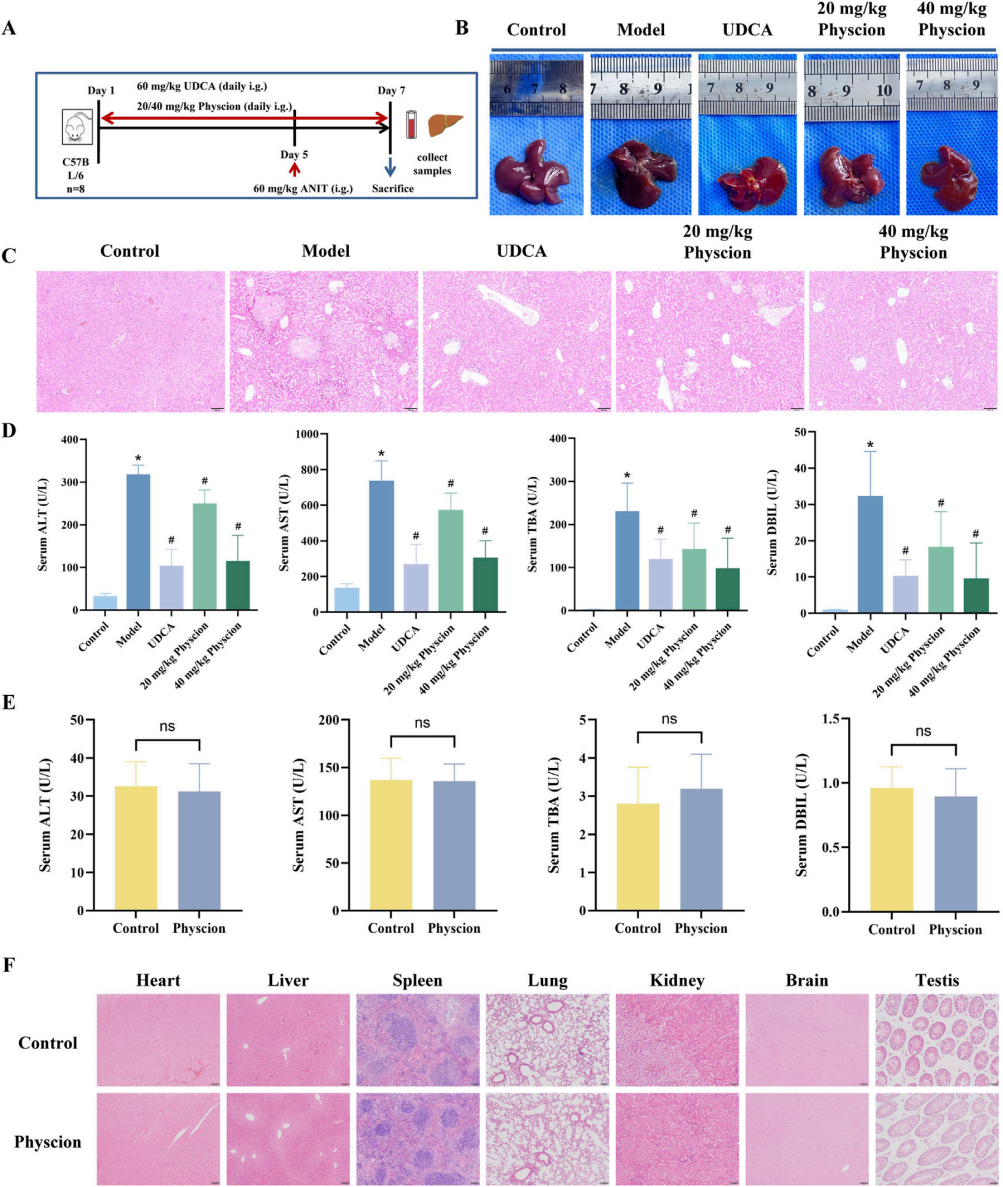
Effect of physcion on ANIT-induced CLI and safety in mice **(A)** Experimental scheme for ANIT-induced CLI and physcion administration. **(B)** Macroscopic appearance of livers from each group. **(C)** Representative images of HE-stained liver sections from each group of mice. **(D)** Serum levels of ALT, AST, TBA, and DBIL. Control, low- and high-dose physcion groups (N = 8), UDCA group (N = 6), and model group (N = 7). **(E)** Serum levels of ALT, AST, TBA, and DBIL after administration of physcion alone at 40 mg/kg. N = 8. **(F)** Representative HE-stained sections of major organs from mice treated with physcion alone. All data are presented as mean ± SD. *P < 0.05 vs. Control, #*P* < 0.05 vs. Model, ns *P* > 0.05 vs. Control.

The experimental protocol for establishing the CLI model and administering physcion was identical to that used for emodin (Figure 7A). During the experiment, one mouse in the model group died, and two mice in the UDCA group died. Compared to the normal livers of the control group, those from the model group appeared yellow with grayish-white spots and fragile margins (Figure 7B). The UDCA and physcion-treated groups showed notable improvements in liver appearance. HE staining further showed that the model group exhibited widespread hepatocyte necrosis, hepatocyte edema accompanied by inflammatory infiltration, fatty degeneration, and congestion in the portal areas (Figure 7C). In contrast, UDCA and physcion treatment significantly alleviated these pathological changes. As shown in Figure 7D, the model group exhibited significantly elevated serum levels of ALT, AST, TBA, and DBIL compared with the control group (*P* < 0.05). Compared with the model group, the levels of these serum biochemical indicators were significantly reduced by UDCA and low- and high-dose physcion (*P* < 0.05). Percentage reductions are provided in Supplementary Table S4. These results indicate that physcion exerts hepatoprotective effects against CLI. Additionally, there were no significant differences in the serum levels of ALT, AST, TBA, and DBIL between the physcion-alone and control groups (Figure 7E, *P* > 0.05). HE staining revealed no observable pathological abnormalities in the major organs, including the heart, liver, spleen, lung, kidney, brain, and testis (Figure 7F).

### 3.8 Regulation of inflammatory factors and the FXR signaling pathway by physcion in CLI mice

**FIGURE 8:**
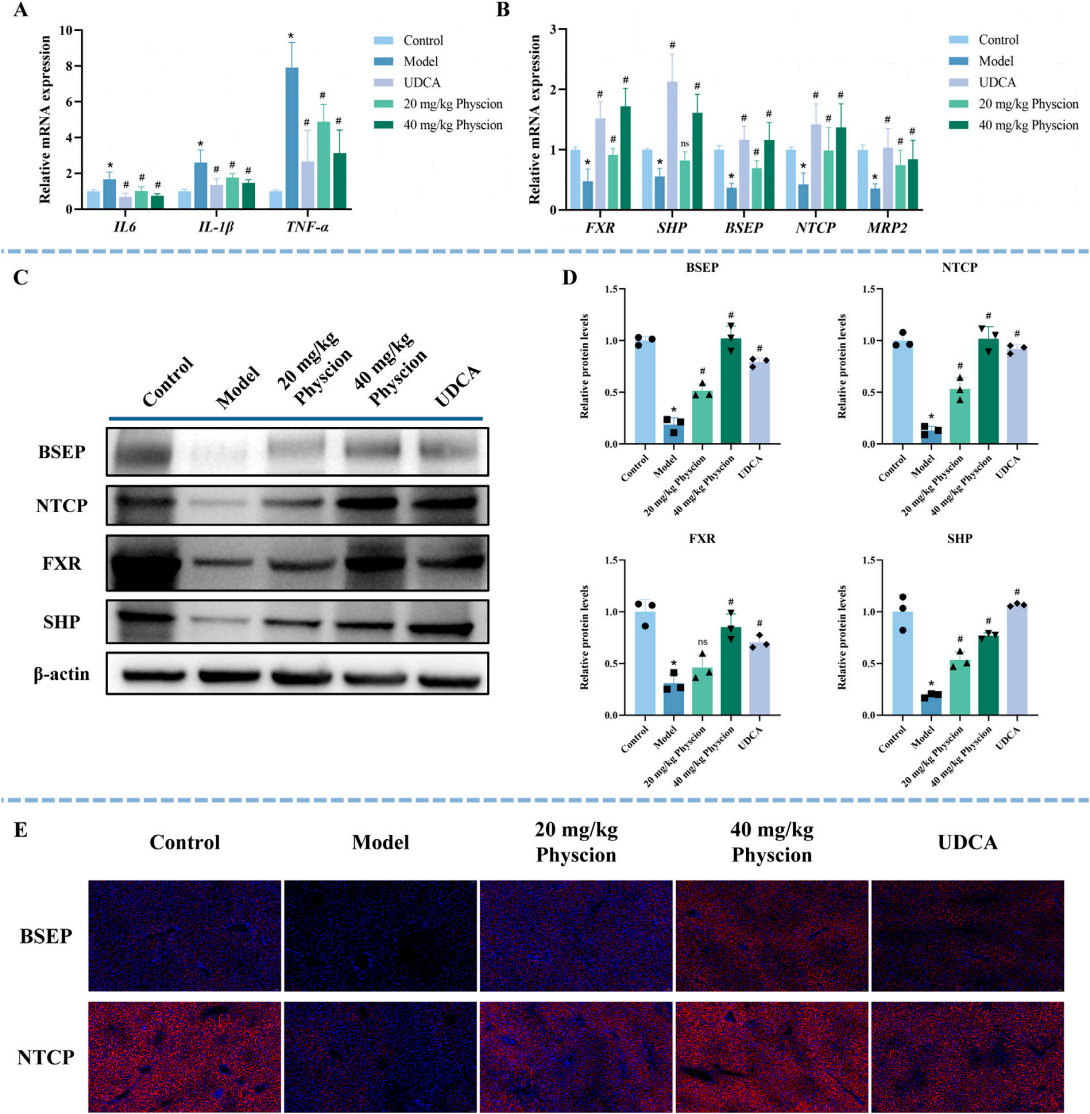
Effect of physcion on inflammatory factors and the FXR signaling pathway in ANIT-induced CLI mice. **(A)** The expression of inflammatory factors (*IL-6*, *IL-1β*, and *TNFα*) was determined by qRT-PCR. N = 5. **(B)** The expression of *FXR*, *SHP*, *NTCP*, *BSEP*, and *MRP2* was determined using qRT-PCR. N = 5. **(C,D)** Representative WB images and quantitative analysis of FXR, SHP, BSEP, and NTCP protein expression in liver tissues. N = 3 independent biological replicates per group. **(E)** Representative immunofluorescence images of BSEP and NTCP (red) in the liver sections. Nuclei are counterstained with DAPI (blue). All data are presented as mean ± SD. *P < 0.05 vs. Control, #*P* < 0.05 vs. Model, ns *P* > 0.05 vs. Model.

To further investigate the mechanism of physcion in protecting mice from CLI, qRT-PCR, WB, and immunofluorescence techniques were performed. The mRNA expression levels of *IL-6*, *IL-1β*, and *TNFα* in the model group were substantially higher than those observed in the control group (Figure 8A, *P* < 0.05), whereas the UDCA and both low- and high-dose physcion groups significantly reduced the expression of these inflammatory factors in a dose-dependent manner (*P* < 0.05).

As illustrated in Figure 8B, compared with the control group, the mRNA expression levels of *FXR*, *SHP*, *BSEP*, *NTCP*, and *MRP2* were significantly decreased in the model group (*P* < 0.05). In contrast, the UDCA and physcion groups markedly upregulated their expression (*P* < 0.05). The quantitative fold changes relative to the model group are summarized in Supplementary Table S6. Consistently, WB results (Figures 8C,D) showed that the expression of FXR, SHP, BSEP, and NTCP in the model group was significantly lower than that in the control group (*P* < 0.05). In contrast, the expression of SHP, BSEP, and NTCP in the UDCA and physcion groups significantly increased (*P* < 0.05). For FXR, a significant increase was observed in the UDCA and high-dose physcion groups, whereas the low-dose physcion group showed only an increasing trend without statistical significance (*P* > 0.05). Immunofluorescence analysis further demonstrated that the expression of BSEP and NTCP was significantly reduced in the model group, while UDCA and physcion treatment dose-dependently upregulated the expression of BSEP and NTCP (Figure 8E). These results suggest that physcion alleviates CLI by suppressing the expression of inflammatory factors and upregulating the expressions of FXR, SHP, BSEP, NTCP, and MRP2.

### 3.9 Effect of FXR inhibition on the hepatoprotective activity of physcion in ANIT-induced CLI

**FIGURE 9:**
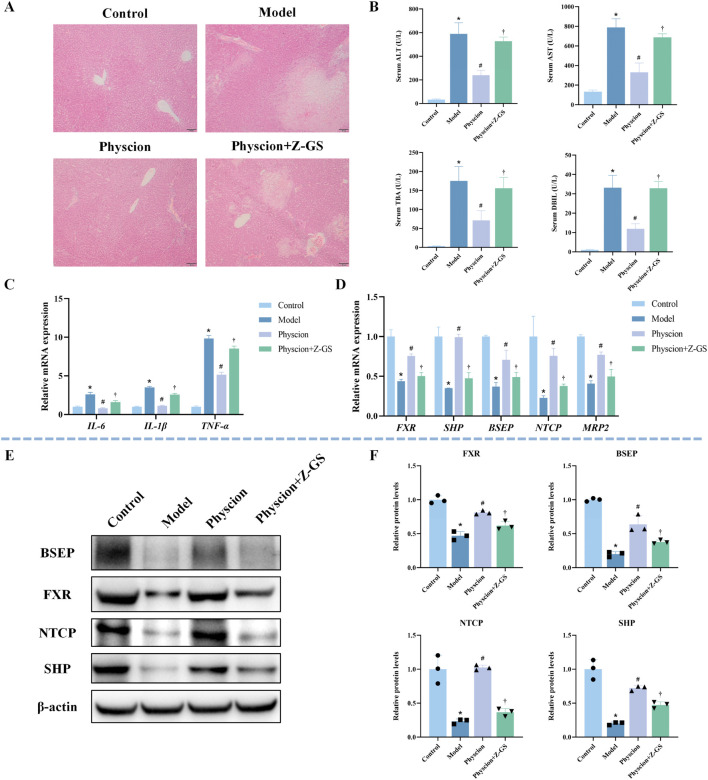
Impact of FXR inhibition on physcion-mediated hepatoprotection and FXR pathway activation in ANIT-induced CLI mice **(A)** Representative images of HE-stained liver sections. **(B)** Serum levels of ALT, AST, TBA, and DBIL. N = 8. **(C)** Hepatic mRNA expression of inflammatory cytokines (*IL-1β*, *IL-6*, and *TNF-α*) was quantified by qRT-PCR. N = 3. **(D)** The mRNA expression of *FXR* and its target genes (*SHP*, *BSEP*, *NTCP*, *MRP2*) was quantified by qRT-PCR. N = 3. **(E,F)** Representative WB images and quantitative analysis of FXR, SHP, BSEP, and NTCP protein expression in liver tissues. N = 3 independent biological replicates per group. Data are presented as the mean ± SD. *P < 0.05 vs. Control; #*P* < 0.05 vs. Model; †*P* < 0.05 vs. Physcion.

As shown in Figure 9A, co-administration of Z-GS markedly attenuated the protective effect of physcion against liver injury. Consistently, Z-GS significantly diminished the ability of physcion to lower serum ALT, AST, TBA, and DBIL levels (Figure 9B). Furthermore, Z-GS notably reversed the inhibitory effects of physcion on the expression of *IL-1β*, *IL-6*, and *TNF-α* (Figure 9C). Z-GS also significantly attenuated the physcion-induced upregulation of *FXR* and its downstream genes *SHP*, *BSEP*, *NTCP*, and *MRP2* (Figure 9D). WB analysis further confirmed that the increased expression of FXR, SHP, BSEP, and NTCP induced by physcion treatment was significantly reduced upon Z-GS co-administration (Figures 9E,F). Collectively, these findings demonstrate that the hepatoprotective effect of physcion against CLI is mediated through FXR activation.

## 4 Disscussion

FXR has emerged as a critical therapeutic target in CLI (Petrescu and DeMorrow, 2021; Trauner and Fuchs, 2022). In this study, FXR was selected as the molecular target, and a comprehensive 2D FXR biochromatography system was established to screen potential FXR-binding compounds from R-PM and P-PM, aiming to elucidate the material basis and underlying mechanism of enhanced efficacy and reduced toxicity following PM processing. The comprehensive 2D FXR biochromatography system was constructed using an MPTS-modified stationary phase. Obeticholic acid (a positive control drug) and tetracycline (a negative control drug) were used to evaluate the effectiveness and specificity of the system. Obeticholic acid is a typical FXR agonist that is widely used in clinical practice (Chapman and Lynch, 2020). The results showed that obeticholic acid exhibited significant retention on the FXR column, with a peak time of approximately 22.5 min, indicating a strong binding affinity for FXR. In contrast, tetracycline eluted near the dead time with no apparent retention, indicating no observable binding to FXR. These findings demonstrate that the comprehensive 2D FXR biochromatography system has good effectiveness and selectivity, supporting its application in subsequent compound screening.

After column validation, the comprehensive 2D FXR biochromatography system was applied to screen for potential FXR-binding compounds from R-PM, S-BSJ-PM, and T-BSJ-PM. Preliminary results indicated that emodin, physcion, aloe-emodin, and TSG exhibited strong retention on the FXR column, suggesting their potential binding to FXR. To confirm these findings, the retention behaviors of corresponding standards were further evaluated. All four compounds showed significant retention on the FXR column, supporting their good binding to FXR. Many studies have demonstrated that processing significantly mitigates the hepatotoxicity of PM while augmenting its hepatoprotective effect (Gu Y. et al., 2022; Huang et al., 2024; Li et al., 2017; Liu et al., 2019; Xue et al., 2020). Previous studies have reported that the content of free anthraquinones increases, whereas that of combined anthraquinones decreases progressively during processing (Qian et al., 2024). Moreover, P-PM elevated the content of gallic acid, emodin, and physcion, and decreased the content of TSG and catechins (Liang et al., 2018; Qian et al., 2024). In this study, UPLC-QTOF/MS analysis revealed that the content of the free anthraquinone compounds emodin and physcion increased significantly after processing, which was consistent with previous studies (Liang et al., 2010; Liang et al., 2018). This initial finding was quantitatively confirmed by HPLC, which verified the significant increase in their concentrations after processing. Evidence from the literature supports the hepatoprotective effects of both emodin and physcion (Bai et al., 2025; Chen et al., 2023; Xie et al., 2019; Yao et al., 2020). For example, one study showed that 50 mg/kg emodin alleviated lipopolysaccharide-induced hepatic inflammation *in vivo* (Xie et al., 2019). In addition, 20 and 40 mg/kg physcion alleviated alcoholic liver fibrosis by inhibiting the HMGB1/NLRP3 pathway (Bai et al., 2025). Based on their increased content after processing and proven hepatoprotective activity, it was preliminarily speculated that emodin and physcion may be the key components responsible for the enhanced hepatoprotection and reduced hepatotoxicity of P-PM.

To further validate the affinity between emodin or physcion and FXR, molecular docking, FAC, and SPR technologies were performed. Molecular docking revealed that the known agonist GW4064 exhibited a high docking score of −16.98 kcal/mol, forming stable interactions within the FXR binding pocket. Both emodin and physcion formed suitable steric complementarity with the binding site of FXR. The docking scores of emodin and physcion with FXR were −7.04 kcal/mol and −6.83 kcal/mol, respectively, suggesting good binding ability to FXR. The K_D_ value is an index used to evaluate the affinity between compounds and proteins. FAC is a chromatographic technique used to calculate K_D_ values (Ng et al., 2007). The breakthrough curves of emodin and physcion at various concentrations demonstrated a clear trend: with decreasing concentrations, the breakthrough times increased. The K_D_ values of emodin and physcion were calculated to be 6.91 μM and 10.89 μM, respectively, indicating strong affinity for FXR. Subsequently, SPR analysis was performed for further validation. GW4064 was used to study the validity and selectivity of the SPR system. The K_D_ value of GW4064 was 0.19 μM, indicating a strong affinity for FXR and demonstrating the validity and selectivity of the SPR system. The K_D_ values of emodin and physcion were 7.54 μM and 30.31 μM, respectively. These results support the conclusion that emodin and physcion exhibit strong affinity for FXR.

To investigate the role and mechanism of emodin and physcion in the hepatoprotective effect of P-PM, CLI models were established in mice via oral administration of ANIT (60 mg/kg). Mice were treated with emodin or physcion at doses of 20 and 40 mg/kg, respectively. For most cholestatic liver diseases, UDCA serves as the standard therapeutic approach (Wagner and Fickert, 2020). Therefore, UDCA was selected as the positive control drug in this study. The model group exhibited significantly elevated serum levels of ALT, AST, TBA, and DBIL compared with the control group. Histopathological examination revealed widespread hepatocyte necrosis. These results confirm the successful establishment of the ANIT-induced CLI model. After treatment with emodin or physcion, liver tissues showed notable improvements in appearance, including color, surface smoothness, and edge clarity. The emodin and physcion groups, at both low and high doses, showed significant reductions in the extent of hepatocyte necrosis, hepatocyte edema, and inflammatory cell infiltration. Moreover, serum levels of ALT, AST, TBA, and DBIL were significantly reduced in a dose-dependent manner after treatment with emodin and physcion. These results indicate that emodin and physcion effectively alleviate ANIT-induced CLI in mice. Furthermore, to assess the potential toxicity, emodin and physcion were each administered alone at a dose of 40 mg/kg. There were no significant differences in serum biochemical parameters between the treated and control groups. In addition, HE staining revealed no observable pathological abnormalities in the major organs, including the heart, liver, spleen, lung, kidney, brain, and testis. Collectively, these results demonstrate that emodin and physcion protect against ANIT-induced CLI in mice without observable adverse effects.

FXR regulates hepatic BA transporters such as BSEP, MRP2, and NTCP to promote BA efflux and limit reuptake, thus preventing intrahepatic accumulation of BAs (Panzitt and Wagner, 2021; Yan et al., 2021). BSEP serves as the primary BA efflux transporter, mediating the export of bile salts from hepatocytes (Vitale et al., 2023). MRP2 transports multiple organic anions out of hepatocytes, thereby lowering their intracellular concentration and alleviating their damage to hepatocytes (Jetter and Kullak-Ublick, 2020). NTCP is responsible for the uptake of BAs from the portal circulation into hepatocytes (Tan et al., 2024). FXR activation not only upregulates the expression of SHP, BSEP, and MRP2, but also downregulates the expression of NTCP (Xiang et al., 2023). The FXR signaling pathway also plays a key role in regulating inflammatory responses (Massafra et al., 2018). Studies have shown that FXR activation can inhibit inflammatory signaling pathways such as NF-κB, thereby effectively suppressing excessive hepatic inflammation (Girisa et al., 2021; Wang et al., 2008). In this study, the results revealed that emodin and physcion significantly suppressed the expression of these inflammatory factors in a dose-dependent manner. Furthermore, ANIT administration markedly suppressed the expression of FXR, BSEP, MRP2, and NTCP, as reported previously (Tang et al., 2024). The downregulation of NTCP suggests that the pathological state itself inhibits hepatic reabsorption of BAs, which could further disturb BA metabolism (Zhang G et al., 2025). Consistently, both qRT-PCR and WB results demonstrated that the expression of FXR, SHP, BSEP, and NTCP was significantly reduced in the model group compared with the control group and was restored following intervention with emodin and physcion. Immunofluorescence results further validated these findings. NTCP upregulation may represent a compensatory mechanism that helps re-establish BA homeostasis. In addition, our findings demonstrated that co-administration of the FXR antagonist Z-GS notably attenuated the hepatoprotective effects of emodin and physcion, along with the upregulation of FXR and its downstream targets. In conclusion, these findings suggest that emodin and physcion exert hepatoprotective effects against CLI by activating FXR, upregulating SHP, BSEP, and NTCP expression, and attenuating inflammatory responses.

## 5 Conclusion

In summary, this study successfully established a novel MPTS-modified comprehensive 2D FXR biochromatography system. Using this method, four FXR-binding compounds were identified from R-PM, S-BSJ-PM, and T-BSJ-PM: emodin, physcion, aloe-emodin, and TSG. The contents of emodin and physcion were found to be significantly increased following PM processing, suggesting that they may serve as the primary compounds responsible for the enhanced hepatoprotection and reduced hepatotoxicity after PM processing. Subsequent pharmacological experiments confirmed that both emodin and physcion exert significant protective effects against ANIT-induced CLI. Mechanistically, these effects were attributed to FXR activation, which led to the upregulation of SHP, BSEP, and NTCP expression and suppression of inflammatory responses. Furthermore, co-administration of the FXR antagonist Z-GS notably attenuated the hepatoprotective effects and the upregulation of FXR and its downstream targets. This study established a novel, efficient, rapid, and accurate comprehensive 2D FXR biochromatography system that is suitable for screening targeted components in TCM and can be extended to research on other TCMs.

## Data Availability

The original contributions presented in the study are included in the article/Supplementary Material, further inquiries can be directed to the corresponding authors.
