# Supplementary material for: Emodin and physcion alleviate cholestatic liver injury by targeting FXR: hepatoprotective components identified in processed Polygonum multiflorum Thunb. using a comprehensive two-dimensional biochromatography system
Source: Front Pharmacol. 2025 Dec 19;16:1706401. doi: 10.3389/fphar.2025.1706401 (PMC12757379; doi:10.3389/fphar.2025.1706401)
Supplement: Supplementary file 1 [file Supplementaryfile1.docx]

Supplementary Material

Supplementary Method

1. Ultra-Performance Liquid Chromatography Quadrupole Time-of-Flight Mass Spectrometry (UPLC-QTOF/MS) Analysis

Chromatographic conditions: Agilent Zorbax SB-Aq column (250 × 4.6 mm, 5 μm); mobile phase: 0.1% formic acid - water (A) and acetonitrile (B). Gradient elution was applied as follows: 0%-2% B (0-2 min); 2%-98% B (2-15 min); injection: 5 μL; flow rate: 0.4 mL/min; column temperature: 40°C; testing time: 15 min.

MS conditions: Q-TOF analysis was performed in positive ESI mode with a mass range of m/z 50-1500. Dry gas: 11 L/min at 350°C; nebulizer pressure: 45 psi; capillary voltage: 3500 V; fragmentor voltage: 100-350 V; skimmer voltage: 60 V; octopole RF: 750 V. Data were processed using MassHunter software.

1. Quantification of Emodin and Physcion by High Performance Liquid Chromatography (HPLC)

Accurate amounts of emodin and physcion standards were separately weighed and dissolved in methanol to prepare a standard stock solution containing 51.025 μg/mL of emodin and 49.460 μg/mL of physcion, respectively. A series of working solutions at different concentrations were then prepared by serially diluting the stock solution with methanol. The sample solutions were prepared using the same procedures as previously described.

Chromatographic Conditions: Column: Agilent Poroshell 120 EC-C18; Mobile phase A: water containing 0.1% formic acid; Mobile phase B: acetonitrile containing 0.1% formic acid. Gradient elution: 0-2 min, 95% A; 2-13 min, 95%-5% A; 13-15 min, 5% A. Injection volume: 5 μL; Flow rate: 0.4 mL/min; Column temperature: 40°C; Total run time: 15 min. Mass Spectrometric Conditions: Analyses were performed using a Q-TOF mass spectrometer operated in full-scan mode over an m/z range of 50-1500 under positive electrospray ionization (ESI) mode. The ESI source parameters were set as follows: drying gas flow rate, 11 L/min; drying gas temperature, 350°C; nebulizer pressure, 45 psi; capillary voltage, 3500 V; fragmentor voltage, 60 V. Reference ions were set at m/z 121.050873 and 922.009798.

Three replicate sample solutions from the same batch were prepared and analyzed under the chromatographic and mass spectrometric conditions described above.

1. Animal Experiments

Emodin/Physcion + Z-guggulsterone (Z-GS): Male C57BL/6 mice (7-9 weeks old) were randomly divided into 4 groups (N = 8): (1) control, (2) model, (3) emodin/physcion (40mg/kg), and (4) emodin/physcion (40mg/kg) + Z-GS (10 mg/kg). The administration volume of all groups was 0.1 mL/10 g. Mice in the control and model groups received 0.5% sodium carboxymethyl cellulose (CMC-Na) by oral gavage once daily for seven consecutive days. On day 5, the control group was given peanut oil, while other groups were orally administered 60 mg/kg α-naphthylisothiocyanate (ANIT) dissolved in peanut oil to induce cholestatic liver injury. Mice in the emodin/physcion and emodin/physcion + Z-GS groups received emodin/physcion (40 mg/kg, orally, once daily) for seven days. In addition, mice in the emodin/physcion + Z-GS groups were intraperitoneally injected with Z-GS (10 mg/kg) once daily, starting 4 h before each emodin/physcion administration, for seven consecutive days. On day 8, all mice were anesthetized, and blood samples were collected from the orbital sinus prior to euthanasia. Body and liver weights were recorded. Serum samples and a portion of the liver tissue were frozen at -80°C, whereas the remaining tissues were fixed in 4% paraformaldehyde for histopathological examination.

Supplementary Figure

Figure S1


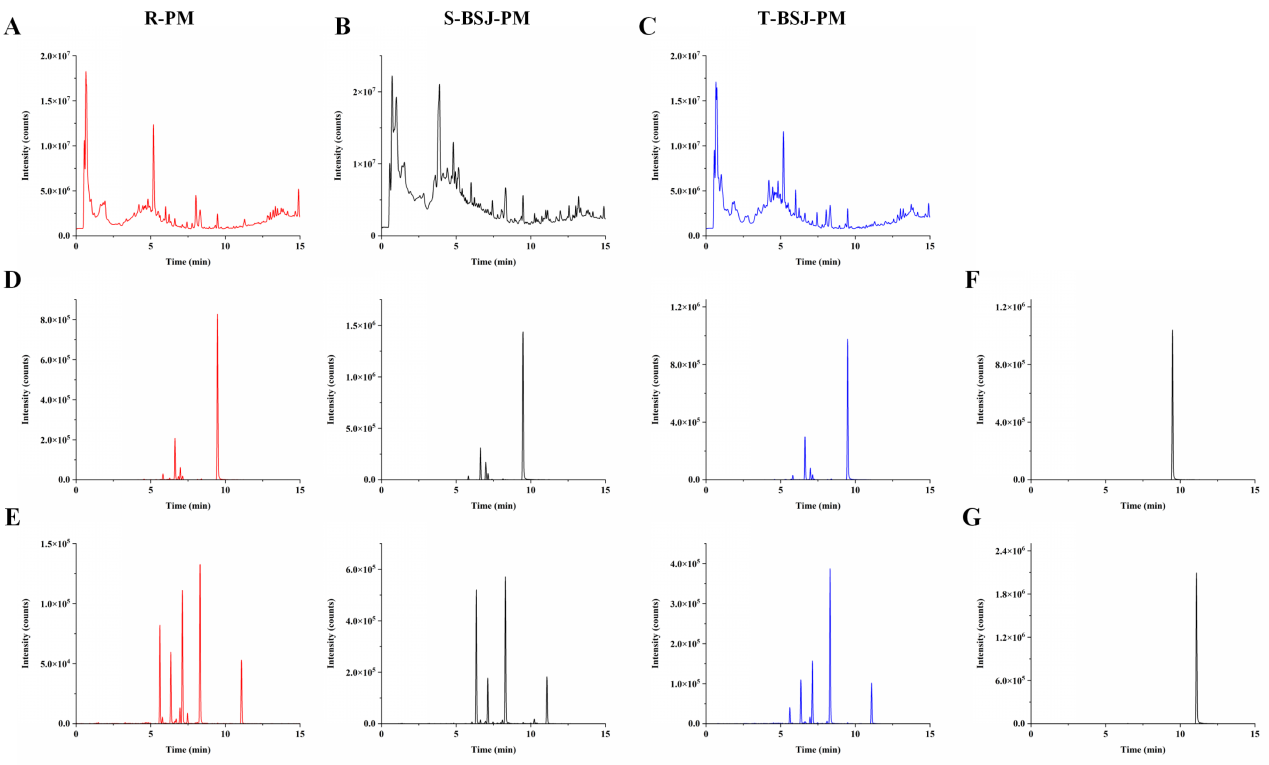


Fig. S1. HPLC analysis of emodin and physcion in different *Polygonum multiflorum* Thunb. (PM) samples.

1. C) Total ion chromatograms (TICs) of the raw PM (R-PM, A), PM steamed with black soybean juice (S-BSJ-PM, B), and PM stewed with black soybean juice (T-BSJ-PM, C) samples. (D) Extracted ion chromatograms (EICs) of emodin from the three PM samples. (E) EICs of physcion from the three PM samples. (F) Representative chromatogram of the emodin standard. (G) Representative chromatogram of the physcion standard.

Supplementary Tables

Table S1. The linear regression equations and linear ranges of emodin and physcion.

| Component | Linear Regression Equation | | Linear Range (μg/mL) | R^2^ |
| --- | --- | --- | --- | --- |
| Emodin | y = 225828x + 159363 | 0.199-51.025 | | 0.9959 |
| Physcion | y = 184352x + 123440 | 0.193-49.460 | | 0.9971 |

Table S2. Concentrations of emodin and physcion in R-PM, S-BSJ-PM, and T-BSJ-PM (μg/mL).

| Component | R-PM | S-BSJ-PM | T-BSJ-PM |
| --- | --- | --- | --- |
| Emodin | 12.244±0.508 | 20.902±3.104* | 17.115±1.209* |
| Physcion | 0.437±0.011 | 3.342±0.194* | 2.109±0.554* |

All data are presented as mean ± SD. N = 3. **P* < 0.05 vs. R-PM.

Table S3. Percentage reductions in serum ALT, AST, TBA, and DBIL levels after emodin treatment (vs. Model, 100%).

| Treatment | ALT | AST | TBA | DBIL |
| --- | --- | --- | --- | --- |
| 20 mg/kg Emodin | 19.16% | 22.05% | 25.98% | 40.94% |
| 40 mg/kg Emodin | 69.79% | 54.88% | 71.01% | 53.55% |

Table S4. Percentage reductions in serum ALT, AST, TBA, and DBIL levels after physcion treatment (vs. Model, 100%).

| Treatment | ALT | AST | TBA | DBIL |
| --- | --- | --- | --- | --- |
| 20 mg/kg Physcion | 21.33% | 23.60% | 38.02% | 43.35% |
| 40 mg/kg Physcion | 63.62% | 59.22% | 57.61% | 70.18% |

Table S5. Percentage changes in the mRNA expression levels of *FXR*, *SHP*, *BSEP*, *NTCP*, and *MRP2* after emodin treatment (vs. Model, 100%).

| Treatment | *FXR* | *SHP* | *BSEP* | *NTCP* | *MRP2* |
| --- | --- | --- | --- | --- | --- |
| 20 mg/kg Emodin | 254.43% | 260.57% | 63.84% | 302.33% | 130.75% |
| 40 mg/kg Emodin | 385.14% | 368.72% | 139.01% | 547.87% | 162.27% |

Table S6. Percentage changes in the mRNA expression levels of *FXR*, *SHP*, *BSEP*, *NTCP*, and *MRP2* after physcion treatment (vs. Model, 100%).

| Treatment | *FXR* | *SHP* | *BSEP* | *NTCP* | *MRP2* |
| --- | --- | --- | --- | --- | --- |
| 20 mg/kg Physcion | 93.89% | 48.52% | 89.54% | 131.66% | 111.78% |
| 40 mg/kg Physcion | 263.72% | 191.01% | 215.33% | 222.27% | 138.06% |

Table S7. Sequence of primers for quantitative real-time PCR analysis.

| ID | Primer name | Sequence of primers (5’-3’) |
| --- | --- | --- |
| 1 | *FXR*-F | GGGATGAGTGTGAAGCCAGCTA |
| 2 | *FXR*-R | GTGGCTGAACTTGAGGAAACGG |
| 3 | *SHP*-F | CCAAGGAGTATGCGTACCTGAAG |
| 4 | *SHP*-R | GCTCCAAGACTTCACACAGTGC |
| 5 | *BSEP*-F | CCTTGGTAGAGAAGAGGCGACA |
| 6 | *BSEP*-R | ATGGCTACCCTTTGCTTCTGCC |
| 7 | *NTCP*-F | CAAACCTCAGAAGGACCAAACA |
| 8 | *NTCP*-R | GTAGGAGGATTATTCCCGTTGTG |
| 9 | *MRP2*-F | TACCAGCGAGTTATCGAAGCGTG |
| 10 | *MRP2*-R | TGCTTCTGACCGCCACTGAGAT |
| 11 | *TNF α*-F | ATGTCTCAGCCTCTTCTCATTC |
| 12 | *TNF α*-R | GCTTGTCACTCGAATTTTGAGA |
| 13 | *IL6*-F | CTCCCAACAGACCTGTCTATAC |
| 14 | *IL6*-R | CCATTGCACAACTCTTTTCTCA |
| 15 | *IL-1β*-F | TCGCAGCAGCACATCAACAAGAG |
| 16 | *IL-1β*-R | AGGTCCACGGGAAAGACACAGG |
| 17 | *GAPDH*-F | GGTTGTCTCCTGCGACTTCA |
| 18 | *GAPDH*-R | TGGTCCAGGGTTTCTTACTCC |
